# Updates on Topical Dyad and Triple Combination Therapies Approved for Acne Vulgaris

**DOI:** 10.7759/cureus.61413

**Published:** 2024-05-31

**Authors:** Alicia Podwojniak, Isabella J Tan, John Sauer, Aarushi Parikh, Bernard A Cohen, Candrice Heath

**Affiliations:** 1 Dermatology, Rowan-Virtua School of Osteopathic Medicine, Stratford, USA; 2 Dermatology, Rutgers Robert Wood Johnson Medical School, New Brunswick, USA; 3 Dermatology, The Johns Hopkins Hospital, Baltimore, USA; 4 Dermatology, Lewis Katz School of Medicine at Temple University, Philadelphia, USA

**Keywords:** tretinoin, azelaic acid, topical treatments, clindamycin, adapalene, combination therapy, benzoyl peroxide, acne vulgaris, acne

## Abstract

Acne vulgaris is a multifaceted disease characterized by inflammatory and noninflammatory lesions. Topical combination therapies offer a multifaceted approach to acne treatment, with synergistic effects and a broad spectrum of action against multiple factors in acne pathogenesis in one single formulation. Clindamycin phosphate/benzoyl peroxide/adapalene, a combination therapy consisting of clindamycin phosphate 1.2%, benzoyl peroxide (BPO) 3.1%, and adapalene 0.15%, is a novel treatment, the only FDA-approved triple combination drug that offers effective treatment of acne vulgaris. This review aims to provide information on clindamycin phosphate/benzoyl peroxide/adapalene and review the literature on combination topical acne medications approved in the United States. This search was conducted on topical combination therapies for acne, their efficacy, adverse effects, and impacts on quality of life with a specific focus on the newly approved clindamycin phosphate/benzoyl peroxide/adapalene and its sub-component dyads, along with other combinations. PubMed, SCOPUS, Embase, Cochrane, and Web of Science databases were searched for publications in 2018-2023. Primary sources were given priority, and secondary sources such as other reviews were considered to supplement any missing information. It was found that various topical dyad and triad combinations exist for acne vulgaris, including adapalene/BPO, tazarotene/clindamycin, clindamycin/BPO, adapalene/clindamycin, topical tretinoin/azelaic acid, topical tretinoin/BPO, and clindamycin phosphate/benzoyl peroxide/adapalene. Dyad and triple combinations represent a promising, convenient solution for acne management, potentially improving patient adherence due to its single formulation. Clindamycin phosphate/benzoyl peroxide/adapalene exhibited significantly high efficacy in treating both inflammatory and noninflammatory lesions, a minimal side effect profile, although no significant changes in quality-of-life measures. Further research is indicated to assess its long-term efficacy and impact on other acne metrics such as cost, scarring, psychosocial implications, and impact on diverse patient populations.

## Introduction and background

Introduction

Acne vulgaris, affecting approximately 9.38% of the global population, poses a significant dermatological concern [1]. It is estimated to affect up to 85% of individuals aged 12-25 in the United States (US), spanning adolescence and early adulthood [2]. Current acne treatments include a spectrum of agents, ranging from topical antibiotics, benzoyl peroxide (BPO), azelaic acid, retinoids, salicylic acid, and clascoterone to oral antibiotics, retinoids, and hormonal medications, each targeting specific aspects of the condition [3]. Several topical combination therapies exist as options to treat acne vulgaris including combinations of antibiotics, retinoids, BPO, adapalene, and salicylic acid [4]. Many of these combinations exist as dyad therapies in which fixed dosages are combined into one formulation, allowing for synergistic efficacy, improved patient adherence and tolerability, and reduced cost.

Complications such as antibiotic resistance, delivery failure, and poor treatment adherence contribute to low monotherapy treatment efficacy. Combating acne pathogenesis has traditionally involved applying multiple individual topical therapies sequentially throughout the day. However, using separate vehicle formulations prevents simultaneous application due to the potential instability of the molecules in their isolated preparations. To address this, combination formulas allow for concomitant application, circumventing potential molecular instability and inactivation. A recent addition to acne therapy, clindamycin phosphate/benzoyl peroxide/adapalene, obtained FDA approval in October 2023 [5]. Comprised of clindamycin phosphate 1.2%, BPO 3.1%, and adapalene 0.15% within a polymeric mesh, this topical gel is tailored for mild to moderate acne. This paper aims to provide information on a newly approved, triple combination acne treatment and review the literature on combination topical acne medications approved in the US. Topical combination therapies offer a multifaceted approach to acne treatment, with synergistic effects and a broad spectrum of action against multiple factors in acne pathogenesis in one single formulation. Hence, they improve patient adherence and address the unmet needs of diverse age groups by offering a safer, more effective, convenient, and potentially affordable solution for acne management.

Methods

This literature review utilized PubMed, SCOPUS, Embase, Cochrane, and Web of Science databases and was conducted in November 2023. Using the National Library of Medicine (NLM) Medical Subject Heading (MeSH) to determine the best selection of potential search terms, the following strings were derived and used: (“Acne Vulgaris” or “acne”) and (“IDP126” or “IDP 126” or “IDP-126 gel”); (“clindamycin”) and (“benzoyl peroxide” or “BPO”); (“clindamycin”) and (“adapalene”); (“adapalene”) and (“benzoyl peroxide” or “BPO”); (“combination therapy”) and (“acne vulgaris” OR acne”).

Inclusion criteria were articles that involved primary data (i.e. randomized controlled trials, cohort studies, retrospective studies, case studies, case series), compelling reviews, human studies only, and pertained efficacy of clindamycin phosphate/benzoyl peroxide/adapalene or other topical dyad therapies to treat acne vulgaris. Articles on combination therapies published between 2018 and 2023 were included, and articles on monotherapy and other aspects of acne were not limited by date. Exclusion criteria were abstracts, articles lacking full text, studies still in progress, and articles that included oral combination therapy.

Full-text appraisal was performed by two reviewers and collected on a data sheet. Each article was analyzed for relevance and proper data reporting and focused on clinical outcomes, efficacy, and adverse effects of the included treatments.

## Review

Types of acne

**Figure 1:**
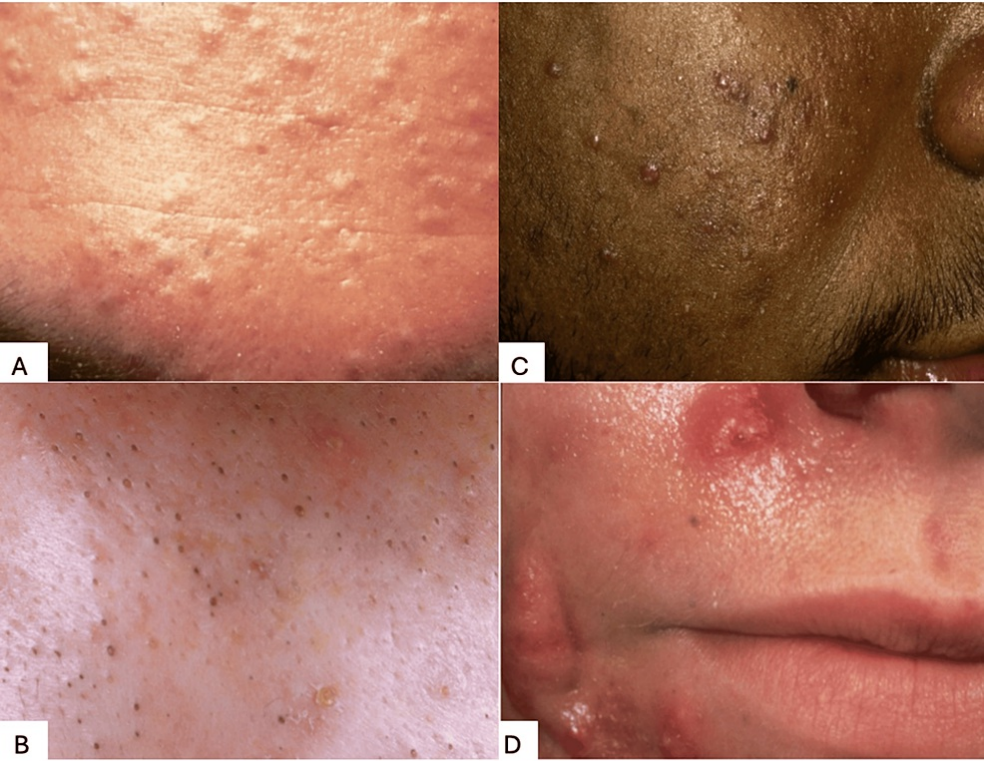
Depiction of various types of acne lesions. (A) Closed comedonal acne in a 25-year-old male; (B) Open comedonal acne in a 16-year-old female; (C) Mixed inflammatory acne in a 19-year-old male; (D) Nodulocystic acne in a 17-year-old male Image Source: Bernard A. Cohen

Acne vulgaris can present as comedones, papules, pustules, and nodules. Various morphologies of acne exist, including comedonal, which can be closed (commonly known as whiteheads) or open (commonly known as blackheads), inflammatory, mixed, or nodulocystic. Comedones are hair follicles that enlarge and fill with keratin, bacteria, and sebum. When open, the surface pigment is oxidized and has a dark appearance, and when closed, the appearance is white. The most severe type, nodulocystic, is made of large inflammatory nodules, often cystic, and has a high predilection to scar [4]. Surrounding erythema and hyperpigmentation may be seen and contribute to the inflammatory appearance. These acne morphologies can be seen in Figure 1.

Acne is commonly classified based on age and severity to guide appropriate treatment approaches. Regarding age, acne is broadly categorized into pediatric acne, affecting preadolescent children, and adolescent/adult acne, which can persistently beyond puberty, or with late-onset. Pediatric acne has been subdivided into four subgroups based on the age of onset-neonatal acne, infantile acne, mid-childhood acne, and preadolescent acne [6]. Persistent adult acne is generally mild and has more inflammatory lesions and fewer comedones than adolescent acne [7]. Acne can also be classified by its severity, especially through evaluating primary lesions or secondary changes to the skin, such as scarring [8]. Severity classification typically ranges from mild, characterized by non-inflammatory lesions such as blackheads and whiteheads, to moderate, involving inflammatory papules and pustules; severe acne encompasses nodules and cysts, often leading to scarring [9]. Individualized management based on age and severity is essential for optimizing outcomes in acne treatment.

Pathophysiology

The multifactorial pathogenesis involves the progression of sebaceous gland hyperplasia, altered follicular growth, bacterial colonization, and inflammation. Follicular hyperkeratinization involves an increase in keratinocyte proliferation and a decrease in the shedding of cells around the openings of sebaceous follicles, forming microcomedones filled with sebum and keratin [10]. Microcomedones develop as precursor lesions for subsequent acne morphologies [11]. A variety of factors, such as diet, psychosocial stressors, comedogenic products, the cutaneous microbiome, and androgen-related pathologies, contribute to the development of various acne forms [1]

Colonization by *Cutibacterium acnes *and additional biota, including *Staphylococcus epidermidis*, *Staphylococcus aureus*, and *Malassezia* are known to contribute to acne development [3]. The activation of innate immunity by *C. acnes *occurs through the expression of protease-activated receptors (PARs), tumor necrosis factor-alpha (TNF-α), and TLRs, along with the keratinocyte production of Interferon gamma (INF-γ), interleukin (IL)-8, IL-12, TNF, IL-1, and matrix metalloproteinases (MMPs), resulting in the inflammation associated with acne lesions [12]. The complex interactions of the cutaneous and gut microbiota and dysbiosis is an emerging area of study believed to contribute, beyond the simple presence of bacteria [13,14]. Differences in microbial diversity are seen among acne patients, with more dysbiosis seen in worse lesions [13]. The presence (or absence) of virulence factors, biofilm production, and overproliferation of certain bacteria are believed to contribute to these diversities [13]. A host’s susceptibility affects the outcome of microbial colonizations, and susceptibility can be impacted by internal and external factors such as genetics, hormones, site of lesions, treatments, and environmental exposures. *C. acnes* has become increasingly resistant to common topical antibiotics such as clindamycin, and thus this previous mainstay of treatment is growing out of favor, with a need for new emerging therapies that minimize this risk. Further, endocrine abnormalities such as premature adrenarche, polycystic ovarian syndrome, thyroid disorders, and prolactin disorders may contribute [12]. Treatments for these causes may involve typical acne therapeutics but should also be focused on treating the underlying cause [4]. 

Mechanisms of topical monotherapies

Topical Retinoids (Adapalene, Tazarotene, Tretinoin, Isotretinoin)

Topical retinoids include adapalene, tazarotene, tretinoin, and isotretinoin. These agents function via inhibition of keratinocyte proliferation and follicular cell turnover. They do not directly have an anti-inflammatory effect but cause a hostile environment for bacterial survival, thus reducing *C. acnes* presence. Further, they inhibit lipoxygenase pathways and release oxygen-free radicals [15]. Adapalene, a synthetic retinoid, available in 0.1% and 0.3% can penetrate the follicle quickly compared to the other agents. It is well-tolerated, effective, and commonly used in combination therapies [16,17]. Tazarotene, available in 0.1% and 0.045%, is suggested to be more effective than adapalene but with a stronger irritation profile, with efficacy in both inflammatory and noninflammatory lesions and oil productions [18]. Tretinoin is available in various dosage forms and has efficacy in comedonal and inflammatory acne forms. These agents show the risk of dryness, erythema, irritant dermatitis, and increased photosensitivity, thus predisposing to burns.

Topical Antibiotics

FDA-approved topical antibiotics often include clindamycin 1%, erythromycin 2-4%, or minocycline 4% to target and reduce colonization of *C. acnes *within the pilosebaceous unit. As macrolide and tetracycline antibiotics, they function to inhibit protein synthesis, and they have anti-inflammatory properties through the reduction of complement activation and granulocyte migration [14,16,19]. They have limited efficacy in non-inflammatory lesions [19], and resistance against them can be developed within weeks, thus posing the need for combination therapy to minimize this risk. Adverse effects are reported to be minimal.

Benzoyl Peroxide

Benzoyl peroxide is among the most widely used, easily available, and inexpensive topical therapies. Available in 2.5%, 5%, and 10% formulations, it functions as an anti-inflammatory and anti-microbial agent that penetrates the stratum corneum to enter the pilosebaceous unit [16]. Its degradation to benzoic acid produces free radicals that damage the cell wall of *C. acnes* and are toxic to yeast [16,20]. It often causes irritation through erythema and dry skin and has also been reported to cause contact dermatitis and swelling [21,22].

Salicylic Acid

Salicylic acid is a beta-hydroxy acid that functions as an acne therapeutic through decreasing skin lipids and its anti-inflammatory properties. One proposed mechanism of decreased sebocyte lipogenesis is via downregulation of the adenosine monophosphate-activated protein kinase (AMPK)/sterol response and element-binding protein-1 (SREBP-1) pathway. Additionally, it is anti-inflammatory via suppression of NF-κB [23]. It has also been shown to dissolve desmosomes within the stratum corneum, leading to decreased adhesion of corneocytes and a comedolytic effect [24]. Dosages range from 0.5%, 30%, and 50% concentrations [23]. Potential side effects include erythema, exfoliation, crusting, dyschromia, and risk of systemic absorption.

Azelaic Acid

Azelaic acid, available in 15% and 20% formulations, is a dicarboxylic acid with antimicrobial and anti-inflammatory actions. Its antibacterial effect is incompletely understood but may be related to the interruption of pH and protein synthesis [25]. Its anti-inflammatory properties stem from the inhibition of cytokines and induction of peroxisome proliferator-activated receptor γ (PPAR γ) [26]. It is not known to confer antibiotic resistance, but common side effects reported are itching and burning [27].

Topical combination treatments

The summary of the studies from the literature search are given in Table 1.

**Table 1:** Summary of literature findings RCT, randomized controlled trial; BPO, benzoyl peroxide; SSA, supramolecular salicylic acid; ADE, adverse drug events

Author	Year	Study Type; Level of evidence	Sample size; Average age (years)	Combination	Outcomes	Adverse drug effects
Lam Hoai et al. [28]	2021	RCT; 1b	n=1029; average age 22.8	Adapalene (0.1%)/BPO (2.5%)	Efficacy in treating acne, showing a favorable safety profile. Many patients discontinued treatment due to ineffectiveness.	Mild dryness, eczema, and acne exacerbation.
Fuchs et al. [29]	2021	Prospective clinical trial; 2b	n=15; average age 22.3	Adapalene (0.1%)/BPO (2.5%)	Demonstrated efficacy to significantly reduce acne lesions and improve acne micromorphology. Decreased hyperkeratinization of follicular borders and reduced intrafollicular keratinous content.	Skin irritation, dryness, and erythema.
Dreno et al. [30]	2018	RCT; 1b	n=67; average age 21.5	Adapalene (0.3%)/BPO (2.5%)	Effective in reducing atrophic acne scars and preventing the formation of new scars.	Mild local skin irritation, most side effects resolved in two to three weeks
Zheng et al. [31]	2019	RCT; 1b	n=31; average age 26	Adapalene (0.01%)/BPO (5%)	Mentions that this combination is used for mild to moderate acne, although showed similar efficacy to 2% SSA	Common side effects included desquamation, dryness, burning, erythema, and pruritus,
Emmerich et al. [32]	2021	Review and expert opinion; 5	n=503; average age 20.5	Adapalene (0.3%)/BPO (2.5%)	Resulted in 73.3% reduction in total acne lesions, a 15.5% reduction in atrophic scar count, and a 16.5% increase in the percentage of subjects achieving a rating “clear” or “almost clear” at 6 months	Local skin irritation, dryness, erythema, contact dermatitis, and burning sensation. This was seen in 4% of participants.
Maiti et al. [33]	2017	RCT; 1b	n=30; average age 21.6	Adapalene (0.1%)/ clindamycin (1%)	>50% drop in lesion count seen in 22% of participants Effectively reduced the total number of facial acne lesions but was inferior to tazarotene/clindamycin.	Mild adverse effects including a burning sensation, itching, and drying of the skin (similar to other group)
			n=30; average age 21.6	Tazarotene (0.1%)/ clindamycin (1.2%)	>50% drop in lesion count seen in 71% of participants Significantly improved inflammatory and noninflammatory lesions compared to Adapalene/clindamycin group	Mild adverse effects including a burning sensation, itching, and drying of the skin (similar to other group)
Hayashi et al. [34]	2018	Phase 4 RCT; 1b	n=169; average age 19.8	Adapalene (0.1%)/ clindamycin (1.2%)	Less favorable, with less efficacy for early treatment of acne vulgaris compared to BPO/clindamycin dyad.	Localized reactions such as dryness, peeling, and burning/stinging.
			n=165; average age 20.3	BPO (3%)/ clindamycin (1.2%)	Greater efficacy for early treatment of acne compared to Adapalene/clindamycin dyad. Reduction in total lesion counts at 2 weeks and inflammatory lesions from 2 weeks onward.	Application site dryness and pruritus.
Mohammadi et al. [35]	2019	RCT; 1b	n=100; average age 18.64	BPO(1%)/ clindamycin (1%)	Nonsignificant reduction in acne lesions with combination use compared to clindamycin alone.	No increases in ADE with combination therapy
Heckman et al. [36]	2019	Prospective cohort; 2b	n=12; average age 29.4	BPO (5%)/ clindamycin(1%)	No significant differences in *Cutibacterium acnes* growth following BPO/clindamycin use	Does not comment on ADE
St. Surin et al. [37]	2020	Case series; 4	n=4; age (30,33, 24,32)	Topical tretinoin (0.05%)/ azelaic acid (15%)	This combination reduced acne lesions and improved hyperpigmentation on the chest, shoulders, and back; limited sample size noted	Well-tolerated without evidence of retinoid dermatitis/ xerosis in treated area
Del Rosso et al. [38]	2023	RCT; 1b	n= 571; age >9 years old	Topical tretinoin (0.1%)/BPO (3%)	Combination therapy was significantly superior to vehicle in both studies (p < .001, p=0.018, respectively)	Treatment was mostly tolerable; local cutaneous reactions of erythema, dryness, pigmentation, and scaling
Stein Gold et al. [39]	2022	Phase 2 RCT; 1b	n = 741; average age 19.5	Clindamycin phosphate/benzoyl peroxide/adapalene (clindamycin phosphate 1.2%, BPO 3.1%, and adapalene 0.15%)	Significant treatment success compared to vehicle gel (p<0.01). Significant reductions in both inflammatory and noninflammatory lesions compared to vehicle gel (p<0.001)	Mild to moderate severity for erythema site pain, dryness, irritation, and exfoliation; no discontinuation of use due to ADE
Eichenfield et al. [40]	2023	Phase 2 RCT; 1b	n = 394; average age 14.9	Clindamycin phosphate/benzoyl peroxide/adapalene (clindamycin phosphate 1.2%, BPO 3.1%, and adapalene 0.15%)	Significant treatment success compared to vehicle gel (p < .001) or any of the dyad combinations (p<0.01). Significant reductions in both inflammatory and noninflammatory lesions compared to vehicle gel (p<0.001). Improvements in acne quality of life scores seen in clindamycin phosphate/benzoyl peroxide/adapalene group	Site pain and dryness were of mild-moderate severity, highest rates in clindamycin phosphate/benzoyl peroxide/adapalene group and adapalene/BPO group, although more in the adapalene/BPO group. Increases in erythema, scaling, itching, burning, and stinging in all groups at week 2 mark. Erythema decreased at week 12 mark in clindamycin phosphate/benzoyl peroxide/adapalene group and increased in all other dyad combinations
Stein Gold, Lain et al. [41]	2022	Phase III RCT; 1b	n=363; average age 20.0	Clindamycin phosphate/benzoyl peroxide/adapalene (clindamycin phosphate 1.2%, BPO 3.1%, and adapalene 0.15%)	Significant treatment success compared to vehicle gel (p<0.01). Significant reductions in both inflammatory and noninflammatory lesions compared to vehicle gel (p<0.001)	Mild to moderate severity for erythema site pain, dryness, irritation, and exfoliation; <4% discontinuation of use due to ADE
Draelos et al. [42]	2023	Phase I and Phase II RCTs; 1b	n=1020; average age 37	Clindamycin phosphate/benzoyl peroxide/adapalene (clindamycin phosphate 1.2%, BPO 3.1%, and adapalene 0.15%)	Study focus on safety profile	No confirmed sensitization or contact dermatitis clindamycin phosphate/benzoyl peroxide/adapalene. ADEs included irritation, dryness, exfoliation, reported as “moderate irritation”. Was significantly less irritating than BPO adapalene dyad (p<0.001)

Adapalene and BPO

The pairing of adapalene and BPO is a recommended treatment for moderate papulopustular acne, highlighting notable efficacy across numerous studies [28-31]. Adapalene, having anti-inflammatory and comedolytic properties, synergizes effectively with BPO, recognized for its antimicrobial and anti-inflammatory effects [28]. The combination significantly reduced acne lesions and improved acne micromorphology by decreasing the hyperkeratinization of follicular borders and reducing intrafollicular keratinous content [29]. However, in one study, the fixed combination of adapalene 0.1% and BPO 2.5% demonstrated a median treatment duration of two months, with a 50% likelihood of discontinuation after three months. Common reasons for stopping included ineffectiveness (52%) and side effects (9%), with controlled acne accounting for a fraction (9%) of cases [28]. Despite potential side effects such as dryness and eczema, adapalene/BPO treatment was generally well-tolerated, aligning with results reported in randomized controlled trials [28].

Furthermore, studies by Emmerich et al. [32] and Dreno et al. [30] focused on using adapalene 0.3%/BPO 2.5% gel as an acne lesion and acne scar treatment. In Dreno et al, the treatment demonstrated efficacy in reducing atrophic acne scars over 24 weeks, preventing scar formation, and reducing existing scars [30]. The incremental increase in efficacy over time emphasized the importance of a more extended treatment duration. While mild skin irritation is usually expected with topical retinoids, most side effects resolve within two to three weeks despite continued therapy. Adjusting treatment regimens during the first four weeks can enhance local tolerability without hampering overall efficacy. Patient-reported outcomes indicated a decline in acne scars and high subject satisfaction, signaling a positive impact on the quality of life for subjects [30]. Similarly, in Emmerich et al., it was found that the adapalene 0.3%/BPO 2.5% combination resulted in a 73.3% reduction in total acne lesions, a 15.5% reduction in atrophic scar count, and 16.5% increase in the number of participants having a rating of “clear” or “almost clear” on the Investigator's Global Assessment (IGA) scale at six months [32]. This study found similar mild adverse effects as seen in Dreno et al [30]. The dual usage of adapalene plus BPO is an effective acne treatment, boasting a generally favorable safety profile.

Adapalene vs. Tazarotene and Clindamycin

A study by Maiti et al. provide insight into the efficacy, safety, and impact on the quality of life associated with the use of adapalene (0.1%) plus clindamycin (1%) or tazarotene (0.1%) plus clindamycin (1%) on acne patients [33]. The combination of tazarotene plus clindamycin proved to be more successful when compared to adapalene plus clindamycin. The reduction in the overall number of acne lesions, the investigator’s static global assessment (ISGA) score, the Global Acne Grading System (GAGS), and the overall acne quality of life (Acne-QoL) score were among the metrics that showed this superiority. Notably, the tazarotene plus clindamycin group demonstrated a more substantial decrease in both inflammatory and non-inflammatory lesions than the adapalene plus clindamycin group. Furthermore, improvements in ISGA scores and Acne-QoL scores were observed in the tazarotene plus clindamycin group [33]. Similarly, when adapalene/clindamycin is compared to BPO/clindamycin it was found to be less efficacious for treating acne vulgaris [34].

Regarding safety, the study revealed a comparable tolerability profile of both regimens with a similar adverse event profile in both groups. The adverse effects were generally mild, including sensations such as burning, itching, and skin dryness. Most importantly, the general finding of the study highlighted how well-tolerated both treatment plans were by the patients [33,34].

Regarding the effect on quality of life, the group that received tazarotene plus clindamycin demonstrated significant improvements compared to the adapalene plus clindamycin group. The improvements were particularly prominent in self-perception and emotional impacts, indicating that the tazarotene-based treatment successfully treated acne lesions and enhanced the patients’ quality of life [33]. This study highlights the superior efficacy of tazarotene plus clindamycin, comparable safety profiles for both regimens and the positive influence of tazarotene on the quality of life of individuals undergoing acne treatment.

BPO and Clindamycin

Hayashi et al. report the findings of a clindamycin phosphate 1.2%/BPO 3% combination gel versus topical adapalene 0.1% gel/clindamycin 1.2% gel in a Japanese, multicenter, randomized parallel-group study [34]. With a sample size of 351 males and females aged 15-45 years, the BPO/clindamycin group had significant efficacy for both total lesions and inflammatory lesion reduction, but no differences were noted for noninflammatory lesions (p = 0.008, p<0.05 respectively). Adverse effects for both treatment groups included dryness, erythema, itching, and stinging. Greater prevalence was noted for the adapalene/clindamycin group although non-significantly. Both treatment groups had high levels of compliance and satisfaction.

Mohammadi et al. report the findings of a double-blind clinical trial of 100 participants, receiving either BPO 1%/clindamycin 1% combination or isolated clindamycin 1% [35]. There were reductions in the average percentage of acne lesions and reduced adverse effects in the combination group, although this finding was non-significant. Heckman et al. report findings of clindamycin and BPO combination therapy regarding BPO’s antibacterial role in preventing bacterial resistance [36]. With a sample of 12 patients, there were no significant differences in the growth of *C. acnes *following combination, isolated BPO or clindamycin, or controlled sites. However, this study has a very limited sample size.

Topical Tretinoin and Azelaic Acid

St Surin-Lord and Miller conducted a small study showing the successful management of truncal acne using a combination of tretinoin lotion 0.05% and azelaic acid 15% foam [37]. Their research revealed a sustained improvement in long-standing, previously treatment-refractory truncal acne among four female African American patients during the follow-up observation period. The treatment regimen resulted in a reduction in acne lesions and improvements in hyperpigmentation on the chest, shoulders, and back. It was well-tolerated without evidence of retinoid dermatitis or xerosis in treated areas. Despite these promising results, the authors maintain a cautious stance, acknowledging the potential limitations of their findings due to the relatively short follow-up duration and small case size.

Topical Tretinoin and BPO

Del Rosso et al. conducted two phase 3 double-blind, randomized, vehicle-controlled studies assessing the efficacy and safety of microencapsulated benzoyl peroxide (E-BPO) and tretinoin cream (E-BPO/T) for the treatment of acne vulgaris [38]. The studies involved a total of 571 subjects with the trials spanning a 12-week treatment period. The group evaluated subjects for various efficacy and safety endpoints during the study at different intervals.

The quantitative results of the study prove the superiority of E-BPO/T over the vehicle cream, a cream containing the same base formulation as the active treatment but without the active ingredients (BPO and tretinoin). The first study found that 38.5% of subjects treated with E-BPO/T achieved IGA success at week 12, compared to 11.5% with the vehicle cream (p<0.001). In the second study, 25.4% of E-BPO/T-treated subjects achieved IGA success, outperforming the 14.7% success rate observed with the vehicle cream (p=0.017). Furthermore, E-BPO/T demonstrated significant reductions in both inflammatory and noninflammatory facial lesions compared to the vehicle cream, substantiated by changes from baseline lesion counts. The safety profile found with the E-BPO/T treatment was tolerable, with the most common local cutaneous reactions being erythema, dryness, pigmentation, and scaling [38].

Deeper dive into newly approved combination therapies

Clindamycin Phosphate/Benzoyl Peroxide/Adapalene

Clindamycin phosphate/benzoyl peroxide/adapalene is a combination therapy consisting of clindamycin phosphate 1.2%, BPO 3.1%, and adapalene 0.15%. Its formulation lacks preservatives and occlusive agents, aims to improve hydration through pH balance [39]. Applied once daily, it addresses acne by targeting inflammation, bacterial growth, and follicular hyperkeratinization. Although this mechanism is not unique to clindamycin phosphate/benzoyl peroxide/adapalene, it has shown strong efficacy, likely through its low side effect profile and high treatment adherence.

Stein Gold et al. describe the results of phase II, a double-blind, multicenter, randomized, 12-week study, in which participants with moderate or severe acne were randomized to clindamycin phosphate/benzoyl peroxide/adapalene, BPO 3.1%/adapalene 0.15% dyad, clindamycin phosphate 1.2%/BPO 3.1% dyad, clindamycin phosphate 1.2%/adapalene 0.15% dyad, or vehicle [39]. With 741 participants, aged greater than nine years old, 52.5% of participants achieved significant improvement in acne lesions, measured by the Evaluator’s Global Severity Score, with clindamycin phosphate/benzoyl peroxide/adapalene compared to all other treatment groups (p<0.001). Significant improvement was also identified in both inflammatory and noninflammatory lesions (p<0.05) [39]. Post hoc analysis by Eichenfield et al. narrowed the sample size to 394 pediatric patients, of which over half were female and over 75% were White [40]. At the 12-week mark, 55.8% of participants receiving clindamycin phosphate/benzoyl peroxide/adapalene reached therapeutic success with significance compared to other dyad groups (p<0.01). At the same time point, the reduction in inflammatory lesions was significantly greater for the clindamycin phosphate/benzoyl peroxide/adapalene cohort (p<0.01). Reduction in non-inflammatory lesions was significantly greater for clindamycin phosphate/benzoyl peroxide/adapalene in all cohorts (p<0.01) except the BPO/adapalene cohort, in which its reduction was nonsignificant (p=0.051) [40]. The results of two phase-III trials reported by Stein Gold et al. again reveal significant clinical efficacy when compared to vehicle gel (p<0.01), along with a significant reduction in both inflammatory and noninflammatory lesions (p<0.001). The time to efficacy was notably shorter in the clindamycin phosphate/benzoyl peroxide/adapalene cohort [41].

Regarding its safety profile, Draelos et al. report the findings of two phase I dermal sensitization studies: Repeat Insult Patch Test (RIPT) and Cumulative Irritation Patch Test (CIPT) [42]. In the RIPT study, which took place over a course of 6-12 weeks, each participant received three patches containing one of three treatments: clindamycin phosphate 1.2%/BPO 3.1%/adapalene 0.15% (clindamycin phosphate/benzoyl peroxide/adapalene) gel, vehicle gel, and saline 0.9% solution, serving as a control. Clindamycin phosphate/benzoyl peroxide/adapalene gel was noted to have a significantly higher irritation score than vehicle or saline (p<0.001); however, it was clinically insignificant [42]. In the CIPT study, lasting 21 days, each participant received five patches coated with one of five treatments: clindamycin phosphate/benzoyl peroxide/adapalene gel, vehicle gel, saline 0.9% solution, sodium lauryl sulfate 0.5%, and BPO 2.5%/adapalene 0.3% gel dyad. Here, clindamycin phosphate/benzoyl peroxide/adapalene again had a significantly higher irritation score than vehicle and saline solutions (p <0.001) but was significantly lower than BPO/adapalene dyad (p<0.001). There were no reports of severe scaling or itching [42]. In the phase II trial results, Stein Gold et al. reported that clindamycin phosphate/benzoyl peroxide/adapalene and BPO/adapalene dyad use resulted in a higher proportion of adverse effects compared to clindamycin with either BPO or adapalene as a dyad or vehicle use [39].These findings were non-significant, and all adverse effects believed to be related to treatment were classified as mild. These included burning, scaling, and stinging. Similar findings were reported in the phase III trials [41]. Five participants in the phase II trial reported dermatitis complications from any of the combinations, including BPO/adapalene (n=2), clindamycin phosphate/benzoyl peroxide/adapalene (n=1), clindamycin/BPO (n=1), clindamycin/adapalene (n=1), although only one of these cases (BPO/adapalene) was believed to be treatment-related [42].

Improvements in quality-of-life measures were highest for the clindamycin phosphate/benzoyl peroxide/adapalene group compared to dyad or vehicle formulations, although these findings were not significant. Particularly, self-perception and emotional impacts were proportionally improved. The self-perception domain assesses the extent to which acne lesions affect feeling self-conscious or feeling dissatisfied with appearance. The emotional domain assesses the extent to which the lesions affect time spent worrying, such as if the lesions will improve, the medication will work, or overall feeling annoyed or bothered by them [39]. Post-hoc analysis by Eichenfield et al. reports improved quality of life in all but one metric for pediatric patients treated with clindamycin phosphate/benzoyl peroxide/adapalene compared to any vehicle or dyad combination [40].

Discussion

The development of acne vulgaris is complex and multifactorial. Treatment options aim to provide optimal appearance outcomes, reduce scar risk and incidence, and minimize psychosocial burden. Topical therapies and maintaining skin hygiene are first-line interventions and refractory cases often progress to systemic therapies [43]. Mono-use antibiotics are less preferred due to resistance from* C. acnes,* and systemic therapies like isotretinoin, although effective, are reserved for severe, refractory cases due to significant adverse effects [44]. Topical combination therapy targets different pathogenesis pathways while minimizing the risk of adverse effects, and is favorable for optimizing patient outcomes [11]. Combining BPO with antibiotic treatment is preferred to combat bacterial resistance, as BPO is known to have bactericidal activity against both the resistant and susceptible strains of *C. acnes* [11,45], though some studies dispute this finding [36]. Adapalene, a topical third-generation retinoid that targets keratinocyte proliferation, is comedolytic and reduces inflammation [46,47]. It is known to retain its functionality when combined with BPO, which also has comedolytic and keratolytic properties, along with antibacterial activity [48]. Topical dyad formulations of clindamycin/BPO and adapalene/BPO have previously been compared, with the former proving to have a higher tolerance and fewer adverse effects [49-51].

A comparison of adapalene/BPO, adapalene/clindamycin, BPO/ tretinoin, and azaleic acid/tretinoin shows multiple drug combinations' efficacy in treating acne [28,30-32,34,28]. Adapalene/BPO effectively reduced papulopustular acne and acne scars, with generally favorable safety profiles. Conversely, tazarotene/clindamycin was found to have better efficacy than adapalene/clindamycin, especially in lesion reduction and improved quality of life [33]. While both treatment plans have similar tolerability, this comparison describes additional effective treatments beyond the well-known adapalene/BPO dyad. Although combinations of BPO and tretinoin are not commonly used due to the potential oxidation of tretinoin by BPO microencapsulation minimized this issue, reducing degradation, optimizing efficacy, and increasing, treatment adherence [38].

Although formulations of acne treatments that contain either BPO with adapalene or BPO with clindamycin exist and are effective, adverse side effects are often noted and can lead to discontinued use. Some studies do not show significant efficacy of these key topical combinations [52,35]. The newly approved combination therapy, clindamycin phosphate/benzoyl peroxide/adapalene, is designed to be applied as a once-daily treatment that targets multiple causes of acne, including inflammation, bacterial growth, and follicular hyperkeratinization, thus providing comprehensive treatment. Its efficacy is apparent through clinical outcomes, reduced inflammatory and non-inflammatory lesions, minimal safety concerns, and improved quality of life. Its gel formulation is designed to minimize irritation as BPO and adapalene are micronized and do not contain alcohol or preservatives, thus minimizing irritation and promoting treatment adherence. One proposed theory of interaction is the possibility that the anti-inflammatory properties of clindamycin provide a neutralizing effect on the tolerability of adapalene and BPO [53,54]. Compared to treatment dyads, clindamycin phosphate/benzoyl peroxide/adapalene was noted to have the quickest time to yield successful outcomes [41]. Low treatment adherence rates for acne vulgaris are often due to prolonged time for visible results and high adverse effect profiles, and often result in the recurrence of lesions, patient dissatisfaction, and increased costs, outcomes to which pediatric patients are particularly susceptible [55]. Adverse effects such as erythema, dryness, and irritation were still present, and increased research is indicated on the persistence or resolution of these adverse effects, along with potential mitigation strategies such as concomitant use of non-comedogenic moisturizers.

Further, for adolescent patients, who often deal with the negative psychosocial effects of acne, quality of life post-treatment is of critical importance for sustaining strong social development [56,57]. Risks of bullying, low self-confidence, avoidance of hobbies, social phobias, depression, and anxiety have been noted among pediatric acne sufferers [58]. Among the various studies, quality of life scores were noted to be improved among the clindamycin phosphate/benzoyl peroxide/adapalene cohorts compared to other treatment options or no treatment controls [40].

Limitations to this review include the recency of FDA approval for clindamycin phosphate/benzoyl peroxide/adapalene and, thus, few studies exploring its long-term effects. Existing studies have disproportionate sample sizes from different racial and ethnic backgrounds. Further research could include effects on these groups, scarring outcomes, increased studies on psychosocial impact, cost analysis, and patient satisfaction.

## Conclusions

The advent of clindamycin phosphate/benzoyl peroxide/adapalene gel represents an innovation in acne treatment, offering a multifaceted approach that targets multiple aspects of acne pathology, especially in reducing both inflammatory and non-inflammatory lesions. This further showcases the advantages of combination therapy, which enhances efficacy and potentially minimizes side effects associated with individual components, enhancing patient adherence due to its once-daily application.

Future studies should be directed toward the efficiency of combination therapies at reducing antibiotic resistance and additional work is needed to assess efficacy in diverse populations. Nonetheless, the application of combined therapies emerges as a promising, convenient, and potentially cost-effective solution, addressing unmet needs in acne management.
